# Presumed acute myocardial damage in a British Shorthair cat with previously diagnosed hypertrophic cardiomyopathy

**DOI:** 10.1186/s13620-026-00348-0

**Published:** 2026-05-23

**Authors:** Sin-Wook Park, Keon Kim, Jong-Won Lee, Yoon-Jung Do, Woong-Bin Ro, Chang-Min Lee

**Affiliations:** 1 Department of Veterinary Internal Medicine, College of Veterinary Medicine and BK21 FOUR Program for Creative Veterinary Science Research Center, Chonnam National University, Gwangju, 61186 Republic of Korea; 2 SKY Animal Medical Center, Gwangju, Republic of Korea; 3 Laboratory of Veterinary Internal Medicine, College of Veterinary Medicine and Veterinary Medical Research Institute, Jeju National University, Jeju, 63243 Republic of Korea; 4 Re-born Animal Medical Center, Busan, Republic of Korea; 5 Division of Animal Diseases and Health, Rural Development Administration, National Institute of Animal Science, Wanju-gun, Republic of Korea

**Keywords:** Echocardiography, Feline, Heart disease, Left ventricular wall thickness, Myocardial disease, Myocardial thickening

## Abstract

**Background:**

Reversible myocardial thickening has been observed in cats and is considered a potential manifestation of acute myocardial injury associated with stress, toxicosis, or systemic illness. However, this phenomenon has not been previously reported in cats with pre-existing hypertrophic cardiomyopathy (HCM).

**Case presentation:**

A 6-year-old castrated male British Shorthair was presented for heart murmur investigation. Based on physical examination, blood analysis, and echocardiography (maximum left ventricular wall thickness (LVWT): 7.5 mm), the cat was diagnosed with HCM phenotype. Three days after the cardiac examination, the cat re-presented with a history of paracetamol ingestion. During hospitalization, the cat developed signs compatible with congestive heart failure. Thoracic radiographs revealed pleural effusion and a diffuse interstitial lung pattern. Echocardiography showed a remarkably increased LVWT (11.4 mm) and an enlarged left atrial diameter. Intravenous fluids were discontinued, and the cat was treated with diuretics, with improvement of its clinical condition. Six months after the cardiac examination, echocardiography revealed a persistent concentric hypertrophy, albeit similar to the initial echocardiographic assessment.

**Conclusions:**

The present case report identified presumed acute myocardial damage associated with paracetamol ingestion in a cat with pre-existing hypertrophic cardiomyopathy. In acutely ill cats, assessment of left ventricular wall thickness should prompt consideration of acute myocardial damage superimposed on chronic cardiac conditions.

## Background

The hypertrophic cardiomyopathy (HCM) phenotype is the most common form of feline cardiomyopathy and has been reported in approximately 15% of screened cats [1, 2]. In a large-scale study, most cats with HCM are asymptomatic at diagnosis, but roughly 30% may develop congestive heart failure (CHF) or cardiovascular death during follow-up [3]. A diagnosis of HCM is made when the left ventricular (LV) end-diastolic wall thickness (≥ 6 mm) is confirmed on echocardiography and other disorders that can cause LV hypertrophy (e.g., hyperthyroidism, systemic hypertension, acromegaly, dehydration) are excluded [1, 4].

Transient myocardial thickening (TMT) is characterised by reversible LV hypertrophy and is thought to reflect an acute myocardial response to injury, such as inflammation or oedema [5–8]. It is considered a potential transient cause of the HCM phenotype in cats [2, 6]. In addition to increased LV wall thickness, TMT may be accompanied by transient left atrial dilation, left atrial dysfunction, and congestive heart failure, although its exact etiology remains unknown [5, 6]. The most frequent antecedent event associated with TMT is surgical/anaesthetic procedures, but others include traffic accidents and diseases that cause pain, and one study revealed that thermal burns may cause myocardial thickening [5, 6, 9, 10].

Paracetamol toxicity in cats leads to oxidative damage, hepatocellular necrosis, and methaemoglobinemia due to their limited capacity for glucuronidation [11, 12]. However, myocardial involvement associated with paracetamol toxicosis has not been clearly documented in cats. This report describes a cat with asymptomatic HCM that developed marked myocardial thickening, left atrial dilation, and CHF within one week of paracetamol toxicosis, followed by a reduction in LV wall thickness (LVWT) and the left atrium to aortic root ratio (LA/Ao).

## Case presentation

**Fig. 1:**
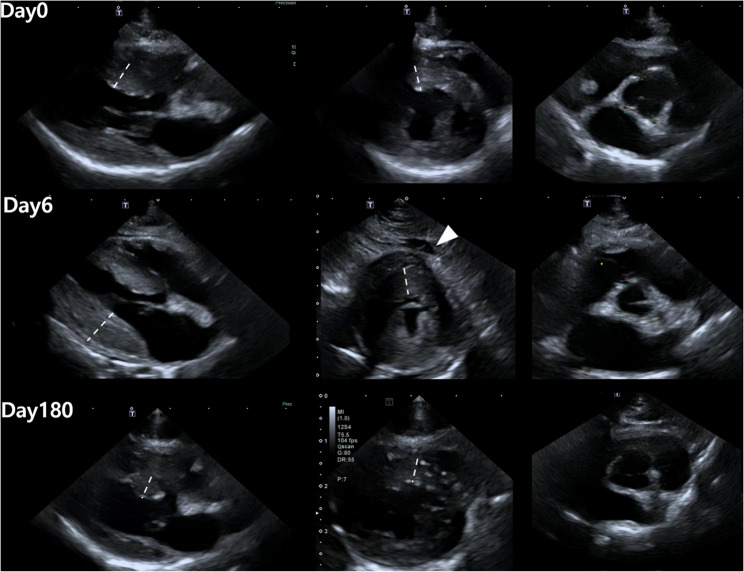
Two-dimensional echocardiographic images of right parasternal views at three timepoints. From the left, a right parasternal long-axis four-chamber view, right parasternal short-axis view at the papillary muscle level, and a right parasternal short-axis view at the aortic valve level are shown. The LVWT was measured from the thickest segment using two- dimensional imaging of the right parasternal long-axis four- and five-chamber views and the short-axis view at the papillary muscle level. Note the drastic increase in left atrial enlargement and left ventricular thickening on day 6. In addition, the dashed line indicates the location of maximal left ventricular wall thickness in each view, and the white arrowhead marks the presence of pleural effusion

A 6-year-old castrated male British Shorthair weighing 5.5 kg was referred to Re-born Animal Medical Center for echocardiography with a history of a heart murmur detected 2 weeks prior to presentation. On physical examination, a grade 3/6 systolic heart murmur (point of maximum intensity: left parasternal) with a regular rhythm and a heart rate of 200 beats/min was detected. The cat’s systolic blood pressure was normal (120 mmHg). Thoracic radiography in ventral and right lateral recumbency revealed no remarkable findings. The cat’s serum NT-proBNP level was elevated at 266 pmol/L (reference range: <100 pmol/L), and the total thyroxine concentration was within the reference range (1.8 µg/dL; reference range: 0.8–4.7 µg/dL). Echocardiographic measurements were performed by one veterinary radiologist and analysed retrospectively by a board-certified internist (Table 1). Left ventricular wall hypertrophy was observed, with a maximum wall thickness of 7.5 mm measured at end-diastole from the interventricular septum using the right parasternal short-axis four-chamber view. Systolic anterior motion (SAM) of the mitral valve was also identified, and the LA/Ao ratio was within the normal range (Fig. 1; Table 1). Twice-daily atenolol (6.25 mg/cat, PO) was prescribed as an individual clinician decision because the presence of SAM raised concern for possible dynamic left ventricular outflow tract obstruction (LVOTO), although overt obstruction was not documented on the initial examination.

**Table 1:** Selective two-dimensional echocardiographic variables at presentation (day 0) and on follow-up echocardiographic examinations

Variable	Unit	Day 0	Day 6	Day 180	Day 450
Heart rate	beat/min	200	120	175	180
Blood pressure	mmHg	130	80	130	140
LA/Ao		1.07	1.79	1.33	1.15
LA FS (%)		28.5	17.4	29.4	32.6
LVIDd	mm	13	8	13.3	12.6
LV FS	%	57.4	62	64.7	58
LVWT Max	mm	7.5	11.4	6.9	7
IVSd MAX (RPLax4C)	mm	7.5	9.5	6.9	6.7
LVFWd MAX(RPLax4C)	mm	6.4	11.4	6.3	6.8
IVSd MAX (RPSaxPM)	mm	7.1	9.4	6.9	7
LVFWd MAX(RPSaxPM)	mm	6.7	9.2	6.1	6.4
AV Vmax	m/s	1.41	2.63	1.10	1.04
PV V max	m/s	0.81	0.56	0.64	0.77
SAM		Yes	Yes	No	No
LVOTO		No	Yes	No	No

*LA/Ao* Left atrium to aortic root ratio, *LA FS *Left atrial fractional shortening, *LVIDd* Left ventricular internal diameter in end-diastole, *LV FS* Left ventricular fractional shortening, *LVWT Max* Maximal left ventricular wall thickness, defined as the greatest measurement obtained from either the interventricular septum or the left ventricular free wall using both right parasternal long-axis and short-axis views at end-diastole, *IVSd Max* Maximal interventricular septal thickness in end-diastole, *LVFWd Max* Maximal left ventricular free wall thickness in end-diastole, *AV Vmax* Peak velocity across the aortic valve, measured using the left parasternal long-axis five-chamber view (m/s), *PV Vmax* Peak velocity across the pulmonary valve, measured using the right parasternal short-axis pulmonary valve view (m/s), *RPLax4C* Right parasternal long-axis four-chamber view, RPSax Right parasternal short-axis papillary muscle level view, *LVOTO* Left ventricular outflow tract, defined in this case as dynamic systolic narrowing of the outflow tract when peak aortic velocity was ≥ 2.5 m/s, *SAM* systolic anterior motion of the mitral valve, indicating anterior displacement of the mitral valve leaflet into the left ventricular outflow tract during systole

Echocardiographic measurements were performed by one radiologist and analysed retrospectively by a single author. No sedation was used for each echocardiogram. All echocardiographic parameters were averaged over three consecutive cardiac cycles. End-diastole was defined as the timepoint immediately following mitral valve closure in long-axis views, and as the point of maximal LV internal dimension in short-axis views

Three days after initial presentation, the cat presented with vomiting, anorexia and lethargy following ingestion of 500 mg paracetamol one day prior. The cat’s vital signs were within normal range (rectal temperature: 38.7 °C, resting respiratory rate: 30 breaths/min, pulse: 150 beats/min, systolic blood pressure: 110 mmHg). The cat was hospitalised (day 0), and blood (which was brown in colour) was collected. A complete blood count (CBC), plasma biochemical, and electrolyte analysis were performed prior to initiating therapy. The cat’s haematocrit (38.9%; reference range: 30.3–52.3%), alanine aminotransferase (ALT: 105 U/L; reference range: 12–130 U/L), and total protein (6.4 mg/dL; reference range: 5.7–8.9 g/dL) were within normal limits. A low plasma creatinine (0.7 mg/dL; reference range: 0.8–2.4 mg/dL), hypophosphatemia (3 mg/dL; reference range: 3.1–7.7 mg/dL), and hypokalemia (3.4 mmol/L; reference range: 3.5–5.5 mmol/L) were also identified. Significant Heinz bodies were found on a blood smear with new methylene blue stain. Therapy was initiated with intravenous fluids (Hartmann solution, 3 mL/kg/h on day 0 and continued at 1 mL/kg/h on days 1–2), N-acetylcysteine (140 mg/kg IV once and continued at 70 mg/kg IV q6h), glutathione (100 mg/cat IV q12h), and vitamin C (30 mg/kg IV q6h), S-adenosylmethionine (50 mg/cat PO q12h), and silymarin (10 mg/kg PO q12h). Including IV medication carrier/diluent volumes, the total IV volume administration rate peaked during the N-acetylcysteine loading dose at 6.5 mL/kg/h (18 mL administered over 30 min). Atenolol was continued throughout hospitalization.

**Fig. 2:**
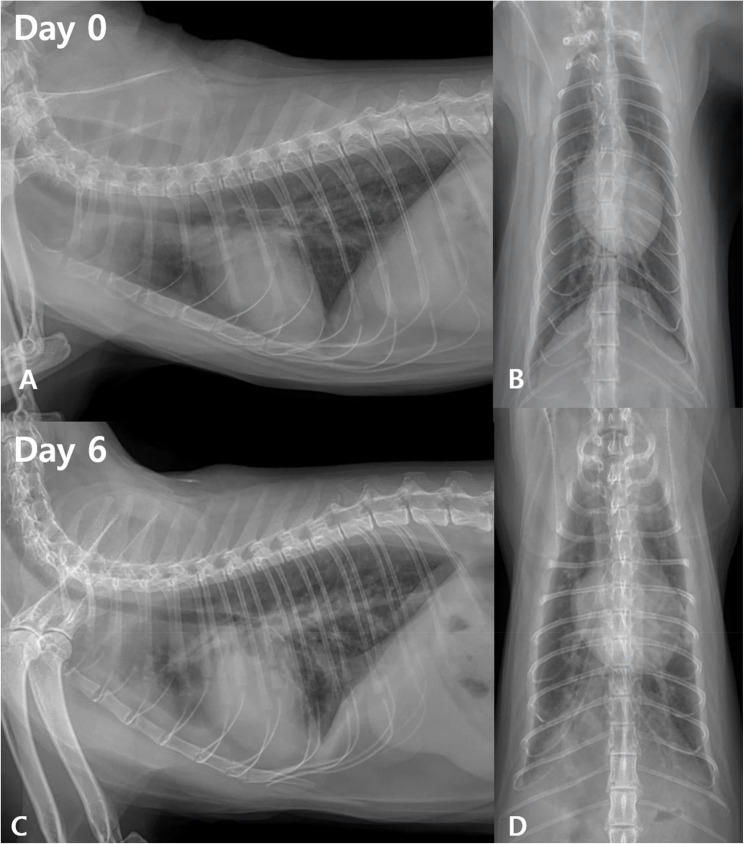
Right lateral (**A**, **C**) and ventrodorsal thoracic radiographs (**B**, **D**). Significant, mild pleural effusion, a mild, localised interstitial pattern with venous congestion were identified. Congestive heart failure was suspected (**C**, **D**)

On day 3, the cat’s hematocrit decreased to 27.3%, and the plasma alanine transaminase levels were above the normal range (193 U/L; reference range: 12–130 U/L) and the total protein concentration was within normal limits (6.3 g/dL). In addition, the cat’s resting respiratory rate increased to 72 breaths/min, and the systolic blood pressure decreased to 80 mmHg. Thoracic radiography in ventral and right lateral recumbency showed increased cardiothoracic ratio, pleural effusion, and a generalised interstitial lung pattern (Fig. 2). Echocardiography revealed mild pericardial and pleural effusion, with increased LV wall thickness (maximum: 11.4 mm) measured from the LV free wall at end-diastole using the right parasternal short-axis four-chamber view. The LA/Ao ratio was increased to 1.79, and left atrial fractional shortening (LAFS) was reduced to 17.4%. LVOTO was also identified [13]. Considering the combination of radiographic and echocardiographic changes, including pulmonary oedema and cardiac enlargement, along with tachypnea and hypotension, the cat was diagnosed with CHF. Intravenous fluid therapy was discontinued, and oxygen therapy was initiated. Furosemide (2 mg/kg IV once and continued at 2 mg/kg PO q12h) and clopidogrel (18.75 mg/cat PO q24h) were also administered.

The cat’s resting respiratory rate decreased to 30 breaths/min on day 5, and radiography showed reduced pleural effusion and improvement of the previously noted interstitial lung pattern. On day 7, the cat’s haematocrit increased to 36.5%, the plasma alanine transaminase levels normalised, and the total protein concentration was 8.0 g/dL, within the normal range. The cat was subsequently discharged with furosemide (2 mg/kg PO q12h), silymarin (10 mg/kg PO q12h), clopidogrel (18.75 mg/cat PO q24h), and atenolol (6.25 mg/cat PO q12h) for 14 days. Follow-up medical treatment was recommended at the local animal hospital.

Six months after the first presentation, the cat presented for repeat echocardiography. Echocardiography revealed a reduced LVWT (LVWT maximum: 6.9 mm) and LA/Ao (1.33), increased LAFS (29.4%) and SAM and LVOTO were not detected. The presumed final diagnosis was acute myocardial damage in addition to pre-existing HCM. A tapering regimen of furosemide was prescribed (1 mg/kg PO q12h), clopidogrel was maintained, and atenolol was discontinued. Fifteen months after the first presentation, the cat presented for repeat echocardiography again and was not receiving cardiac medications, which had been discontinued by the clinician following clinical improvement. Echocardiography revealed similar LVWT (maximum: 7 mm), LA/Ao (1.15), and LAFS (32.6%). SAM and LVOTO were not detected. The owner reported that the cat was doing clinically well.

## Discussion

This report describes a 6-year-old British Shorthair cat with pre-existing HCM that developed presumed acute myocardial injury following paracetamol ingestion. The episode was associated with a transient increase in myocardial wall thickness, a mild increase in LA/Ao, and clinical signs suggestive of CHF. Over time, the HCM phenotype persisted, but myocardial thickness and LA/Ao had returned to approximately baseline values at the 6-month follow-up, and this improvement was sustained at 15 months. The cat remained clinically stable without signs of CHF, even after discontinuation of cardiac medications, suggesting that long-term pharmacologic management may not be required in selected cases where CHF is precipitated and/or exacerbated by transient, reversible factors, including increased preload during IV therapy and beta-blockers, such as volume overload, anaemia, or drug-induced myocardial injury, rather than progression of underlying HCM.

The pathophysiology causing the acute and reversible myocardial thickening in cats remains unclear. Similar cases of acute and reversible myocardial thickening have been reported in cats, often referred to as TMT, which is thought to reflect myocardial oedema secondary to stress, inflammation, or systemic insults [5, 6]. One proposed mechanism involves myocardial oedema caused by catecholamine surges in response to physical or systemic stressors [9, 14, 15]. Prior reports have described similar myocardial changes in association with events such as anaesthesia, trauma, thermal burns, sepsis, and infections including toxoplasmosis and bartonellosis [5–7, 14, 15]. However, in some cases, no specific trigger was identified [5, 6]. In the present case, paracetamol toxicosis, along with intravenous fluid administration and acute anaemia, may have contributed to haemodynamic alterations and myocardial structural changes. Although infectious causes such as toxoplasmosis were not definitively excluded, the cat was strictly housed indoors, making such infections less likely [14].

The clinical progression—transient LV wall thickening, development of CHF, and reduced left atrial contractility—was followed by resolution of these abnormalities on serial re-evaluation, supporting the interpretation of presumed acute myocardial damage superimposed on previously diagnosed HCM [5, 6]. While these findings resemble previously reported cases of TMT, the diagnosis was not applied here because the cat had pre-existing LV hypertrophy consistent with HCM, and the myocardial thickening did not represent a return to a normal wall thickness [6, 15].

Similar reversible myocardial thickening has been described in humans with Takotsubo cardiomyopathy (TTC), a stress-associated condition characterised by transient myocardial dysfunction and myocardial oedema [16–18]. Although TTC is not directly translatable to veterinary medicine, it may offer a conceptual parallel. TTC can result in LVOTO, and when superimposed on HCM, may lead to acute heart failure [19, 20]. In this case, the cat exhibited LVOTO and hypotension, possibly reflecting the combined impact of acute myocardial damage and structural heart disease. Serial echocardiographic assessments were essential to document the dynamic changes and clinical recovery.

Atenolol was initiated at presentation as an individual clinical decision based on the presence of SAM and concern for possible dynamic LVOTO, although overt obstruction was not documented on the initial echocardiogram. In human medicine, beta-blockers may be used in cases of human Takotsubo syndrome complicated by LVOTO to alleviate obstruction [19, 21]. However, the use of beta-blockers in the presence of CHF remains controversial in veterinary medicine. Beta-blockers can reduce cardiac output and worsen congestion due to negative inotropy and chronotropy. While long-term outcomes of atenolol in preclinical HCM have shown no clear benefit [22], its safety and efficacy in feline decompensated heart failure are not well established. In this case, CHF progressed during therapy, suggesting that the negative inotropic effects of atenolol may have outweighed the potential benefits of rate control and gradient reduction. In retrospect, withholding beta-blockers or discontinuing it earlier upon the onset of CHF might have been more advantageous.

Paracetamol exposure may lead to cellular injury and death of hepatocytes, and ingestion of even small amounts can be a substantial risk for the cat [23, 24]. The common clinical signs are anorexia, dullness, facial and paw oedema, muddy mucous membranes, respiratory distress, and haematuria [25]. In addition, oxidative damage may develop with the formation of methaemoglobin that causes anaemia [11]. Moreover, myocardial infarctions, CHF, cardiac arrhythmias, pericarditis, and myocarditis due to paracetamol toxicity have been reported in humans [26, 27]. Potential causes of cardiotoxicities are oxidative stress and decreased sulphydryl groups that may lead to cardiac cell death [26]. In addition, one report demonstrated that TTC developed with paracetamol toxicity, and the echocardiographic findings of this patient were ventricular wall thickening and decreased LV systolic function [28]. There is no report of such cardiotoxicity in cats, and one study reported that pulmonary oedema developed in a cat with paracetamol exposure, but echocardiography was not performed [29]. Since the exact mechanisms of acute myocardial damage remain unclear, this case suggests the possibility of paracetamol-induced myocardial injury that may lead to reversible myocardial thickening.

The main limitation of this report is that cardiac biomarkers, inflammatory biomarkers, and stress biomarkers were not analysed. The second limitation is that fluid analysis of the pleural effusion was not performed, and underlying infectious diseases were not ruled out. In addition, an electrocardiogram was not conducted in this case. Finally, the follow-up interval was relatively long, and the process of normalization of the myocardial wall was not confirmed. Despite these limitations, this case is meaningful as there was reversible myocardial hypertrophy after paracetamol ingestion in this cat with asymptomatic HCM.

## Conclusion

This report described acute myocardial thickening and CHF in a cat with pre-existing HCM followed by partial resolution of echocardiographic abnormalities on serial re-evaluation. Potential contributing factors in this case included paracetamol toxicosis, hospitalisation-related stress, intravenous fluid administration, acute anaemia, and possible effects of beta-blocker. These findings highlight that, in cats with underlying HCM, an acute increase in left ventricular wall thickness may reflect a superimposed transient myocardial process rather than progression of the underlying cardiomyopathy alone. This case also suggests that long-term cardiac medication may not always be necessary when CHF develops in association with a transient myocardial process and follow-up echocardiography shows subsequent improvement.

## Data Availability

No datasets were generated or analysed during the current study.

## Abbreviations

CHF: Congestive heart failure
HCM: Hypertrophic cardiomyopathy
LA: Left atrium
LAFS: Left atrial fractional shortening
LV: Left ventricular
LVOTO: Left ventricular outflow tract obstruction
LVWT: Left ventricular wall thickness
SAM: Systolic anterior motion
TMT: Transient myocardial thickening
TTC: Takotsubo cardiomyopathy
